# Impact of continuous predator threat on telomere dynamics in parent and nestling pied flycatchers

**DOI:** 10.1007/s00442-019-04529-3

**Published:** 2019-10-14

**Authors:** Tiia Kärkkäinen, Pauliina Teerikorpi, Bineet Panda, Samuli Helle, Antoine Stier, Toni Laaksonen

**Affiliations:** 1 Department of Biology, Section of Ecology, University of Turku, Turku, Finland; 2 Department of Biology, Section of Genetics and Physiology, University of Turku, Turku, Finland; 3 Institute of Biodiversity, Animal Health and Comparative Medicine, University of Glasgow, Glasgow, UK; 4 Natural Resources Institute Finland (LUKE), Helsinki, Finland

**Keywords:** Ageing, Birds, Life history, Predator–prey, Trade-off

## Abstract

In addition to direct mortality, predators can have indirect effects on prey populations by affecting prey behaviour or physiology. For example, predator presence can increase stress hormone levels, which can have physiological costs. Stress exposure accelerates the shortening of telomeres (i.e. the protective caps of chromosomes) and shorter telomeres have been linked to increased mortality risk. However, the effect of perceived predation risk on telomeres is not known. We investigated the effects of continuous predator threat (nesting Eurasian pygmy owl *Glaucidium passerinum*) on telomere dynamics of both adult and partially cross-fostered nestling pied flycatchers (*Ficedula hypoleuca*) in the wild. Females nesting at owl-inhabited sites showed impaired telomere maintenance between incubation and chick rearing compared to controls, and both males and females ended up with shorter telomeres at owl-inhabited sites in the end of chick rearing. On the contrary, both original and cross-fostered chicks reared in owl sites had consistently longer telomeres during growth than chicks reared at control sites. Thus, predation risk may cause a long-term cost in terms of telomeres for parents but not for their offspring. Predators may therefore affect telomere dynamics of their preys, which could have implications for their ageing rate and consequently for population dynamics.

**Electronic supplementary material:**

The online version of this article (10.1007/s00442-019-04529-3) contains supplementary material, which is available to authorized users.

## Introduction

Predators can affect prey populations either by killing the prey (i.e. lethal or direct effects), or by changing the behaviour and/or physiology of the prey (indirect or non-lethal long-term effects), which may later influence prey demography via reduced fecundity or survival (Boonstra 2013; Clinchy et al. 2013). Traditionally, studies on predator–prey interactions have paid less attention to the role of indirect effects than to the direct effects even though their impact on prey population dynamics (through, for example, energy intake and/or survival) may be even stronger than that of direct effects (Preisser et al. 2005). To date, the studied non-lethal effects include, for example, reduced clutch size and impaired foraging of the prey individuals (Brown et al. 1988; Travers et al. 2010; Zanette et al. 2011). High continuous predation pressure has also been shown to increase glucocorticoid stress hormones, as well as stress protein levels of adult individuals in several studies (Scheuerlein et al. 2001; Clinchy et al. 2004, 2011; Thomson et al. 2010; Sheriff et al. 2011).

Continuous predator presence may also affect the developing offspring. For example, perceived predation risk experienced by the parents can lower offspring survival (Zanette et al. 2011) and cause elevated transfer of maternal stress hormones to the developing young (Saino et al. 2005; Sheriff et al. 2010). Such transfer might help to produce offspring that are better adapted to stressful environments as shown in snowshoe hares (Sheriff et al. 2010). However, several studies have reported negative effects of maternal glucocorticoids on offspring development, e.g. reduced growth rate (Saino et al. 2005) and altered immune function (Rubolini et al. 2005). Chronic exposure to stress/high glucocorticoid levels can also lead to oxidative stress (imbalance between free radicals and antioxidant defences) (Costantini et al. 2011; Marasco et al. 2017), and accordingly, predator cues have been shown to induce an oxidative stress response in an amphibian species (Pinya et al. 2016). Additionally, parents may reduce their provisioning rates when predators are present (Tilgar et al. 2011; Zanette et al. 2011; but see Hakkarainen et al. 2002; Thomson et al. 2010). As a consequence, the developing offspring may suffer from nutritional stress, which has been shown to lead to increased oxidative stress (Jennings et al. 2000). Importantly, oxidative stress has been shown to accelerate the rate of telomere shortening (Reichert and Stier 2017), which may increase mortality risk (Wilbourn et al. 2018).

Telomeres are highly conserved and specialized, protective non-coding areas at the ends of eukaryotic chromosomes. Telomeres shorten with every cell division due to the end-replication problem and their particular sensitivity to oxidative stress (von Zglinicki 2002; De Lange et al. 2006; Reichert and Stier 2017). Even though the enzyme telomerase can restore telomere length, telomeres shorten with age in most cases (see Stier et al. 2015b for a review in birds), since telomerase expression is usually repressed or minimal in somatic tissues of most endotherms (Gomes et al. 2010). When telomeres reach a critically short length they become dysfunctional, which leads to replicative senescence, apoptosis or genomic instability (De Lange et al. 2006). Considering the important role of the telomeres in cellular ageing and the fact that short telomeres have been linked to increased mortality risk in human and non-model vertebrates (Cawthon 2002; Wilbourn et al. 2018; but see Simons 2015), telomere shortening has been suggested to underlie ageing at the organismal scale (Monaghan and Haussmann 2006). However, whether there is any causal link between telomeres and ageing remains controversial (Young 2018), although short telomeres have been associated with survival and mortality in several passerine species (Barrett et al. 2013; Salmón et al. 2017; Eastwood et al. 2019). Nevertheless, telomere length has been suggested to act as an indicator of future survival prospects, but also as a marker of cumulative stress exposure (Monaghan 2014; Angelier et al. 2017). Accordingly, chronic psychological stress has been associated with shorter telomeres from humans to laboratory mice and wild birds (Epel et al. 2004; Kotrschal et al. 2007; Herborn et al. 2014; Meillère et al. 2015), and prenatal stress exposure has also been shown to result in shorter telomeres later in life (Haussmann et al. 2012). One of the most important and frequent stressors for wild populations is predator threat, but the role of predators in shaping the telomere dynamics of their prey has not yet been investigated (Angelier et al. 2017).

Here, we studied the effects of continuous predator threat on telomere dynamics in the European pied flycatcher (*Ficedula hypoleuca*) during breeding. To this end, we installed nest boxes for flycatchers close to Eurasian pygmy owl (*Glaucidium passerinum*) nests and in similar control sites. To study the telomere dynamics of the nestlings, we conducted a partial cross-fostering experiment to control for potential differences in the original quality of the chicks at different sites. We also examined telomere shortening (females) and length in the end of nestling period in the parents; for them the environment could not however be manipulated, but was the one in which they themselves settled to breed. We predicted that the continuous predator threat could lead to increased rate of telomere shortening in chicks and adults at owl-inhabited sites, and thus to shorter telomeres at the end of growth and chick-rearing periods, respectively. Since early-life telomere dynamics could be influenced by the rate of development (Stier et al. 2015a; Vedder et al. 2018), we also investigated the effect of predation risk on chick growth rate.

## Materials and methods

### Study species

The pied flycatcher is a small, cavity-nesting, long-distance migratory passerine that breeds in most of Europe and western Siberia, and winters in sub-Saharan Africa. In Finland, pied flycatchers arrive to the breeding grounds in May (Lundberg and Alatalo 1992). At their arrival, the nesting predators have already settled down in the so-called predation risk landscape (Thomson et al. 2006). One of the main predators of small passerine birds in the boreal forests is the pygmy owl (Kellomäki 1977). The pygmy owl poses a risk to pied flycatchers, which is seen for instance in the avoidance of breeding in the vicinity of the owl (Morosinotto et al. 2010). In another passerine, the presence of a pygmy owl also leads to higher antioxidant enzyme activity, which could be reflective of increased oxidative stress (Morosinotto et al. 2018). While the pygmy owl is not the only predator of pied flycatchers, as a diurnal owl and a central place forager it poses a great risk to them (Kellomäki 1977; Morosinotto et al. 2010). Thus, pygmy owl’s presence/absence would most arguably contribute significantly to the overall predation risk perceived by the pied flycatchers.

### Field work

Data were collected in 2017 during breeding season in a study area of ca. 500 km^2^ north of the city of Turku in Southwest Finland. Before the arrival of pied flycatchers, we inspected 195 pygmy owl boxes to locate pygmy owl nests. The owl nest boxes were distributed so that there was approximately one per km^2^ in the forested parts of the area. At the beginning of the experiment, there were 11 active pygmy owl nests on different sites. Accordingly, we chose 11 control sites, minimum of 1.1 km from the nearest owl site. If possible, we picked control sites where owls had been breeding previously but were not currently breeding, to confirm that the habitat was suitable for pygmy owls (5 out of 11 sites). The other six sites had a pygmy owl box that had never been occupied by an owl, but the forest patches were similar mature forests dominated by spruce and pine to those occupied by the pygmy owls, and they were distributed over the same area as the owl nests. For the experiment, we installed eight nest boxes suitable for pied flycatchers (17 × 17 × 28 cm with entrance hole Ø of 3.2 cm) in the vicinity of each pygmy owl nest box at both owl-inhabited and at control sites. The basic design of spatial configuration of the boxes was always the same: the boxes were 60–90 m away from the pygmy owl nest and ca. 30–40 m from the nearest neighbouring box. The distance to the pygmy owl nest was decided on the basis of previous studies by Morosinotto et al. (2010) and Moks et al. (2016), both of which indicated that flycatchers are aware of the predator presence at this distance from their nest site.

In late May, we checked all the nest boxes to monitor the species inhabiting the boxes. The boxes inhabited by pied flycatchers were thereafter checked every 4 days to monitor the starting date of egg laying and the final clutch size. Once egg laying was completed, we estimated the start of incubation from final clutch size. 10–11 days (average ± SE = 10.7 ± 0.5 days) after the start of incubation, incubating females were caught, weighed and their wing length was measured and a blood sample (50–75 µl) was taken from the wing vein to assess the initial telomere length (from 7 nests in 6 control sites and 11 nests in 8 owl sites). When their nestlings were 10 days old (average ± SE = 13.3 ± 0.5 days after first sampling), the females were caught again, measured, and another blood sample was collected (we were unable to re-catch one female from an owl site). At this time, males were also captured (from 6 nests in 6 control sites and 11 nests in 7 owl sites), measured and sampled. Blood samples were taken with non-heparinized capillary tubes, diluted in 125 µl of PBS and kept chilled while in the field. At the end of the day, the blood samples were stored at − 80 °C.

We estimated the hatching date from the date of the last laid egg. Nest boxes were checked a day before the estimated hatching day and every day after that until the hatching. Chicks were partially cross-fostered between owl and control sites when they were 3 days old (hatching day = day 0). The cross-fostering study design enables distinguishing genetic/prenatal and environmental effects on a trait by placing full siblings in different rearing environments (Merilä 1996). Cross-fostering nest pairs were matched according to hatching date and original clutch size. On average, pied flycatcher chicks at owl sites hatched later than control chicks due to delayed nest construction and later egg laying, and for this reason five of the owl site nests could not be matched with a control nest on the original sites. We thus had to resort to five additional control site boxes that were out of our study area, to complete the cross-fostering. Only chicks (not adults) from those sites were used in the analyses. Matching the cross-fostering pairs according to the hatching day diminishes the differences in environmental conditions experienced by the chicks and ensures that the chick-rearing period is the same for adults from both control and owl sites despite the general later hatching of the owl site chicks. If there were eggs in the nest that had not hatched by day 3, extra chicks were brought to the nest from extra nests hatched on the same day, to match the original clutch size. If a suitable extra chick was not found, no chicks were added (in four out of ten cases). Extra chicks were not sampled. Two to three chicks (depending on the brood size) were picked randomly and exchanged between the nest pairs, while the rest of the chicks remained in their original nest throughout the study (sample size for chicks per treatment C–C = 33, C–O = 36, O–O = 31, O–C = 36; treatment = original site-rearing site, c = control, o = owl site; cross-fostered chicks are in groups C–O and O–C and those remaining in their own nests in groups C–C and O–O). All exchanges happened within 5 days. During the cross-fostering the chicks were made identifiable by removing gently the feather tufts either from the head or the back. When chicks were 5 days old, they were ringed, weighed and a first blood sample (about 35 µl) was taken. Blood samples were diluted in 65 µl of PBS and kept chilled until stored at − 80 °C at the end of the day. Because less blood was taken from chicks than from adults, PBS volume for chick samples was lower to standardize the dilution factor. When the chicks were 12 days old (2–3 days before fledging), they were weighed and sampled for the second time.

We determined box occupancy, average date of first egg laid, average clutch size, average number of nestlings and fledglings and mean parental body mass between control and owl sites (Table 1). As suggested by Morosinotto et al. (2010), pied flycatchers may have actively avoided breeding near pygmy owls, as box occupancy at owl site was little more than 30% compared to almost 80% at control sites. On average, females at owl sites started egg laying 1 day later than control-site females, but despite of that we observed no significant differences in average clutch size and number of nestlings or fledglings between control and owl sites (Table 1). Adults at control sites were a little heavier than owl site adults, although the differences were not statistically significant.

**Table 1:** General breeding characteristics between control and owl sites

	Control site	Owl site	*p* value
Box occupancy %	77.8	32.1	
Average date of first egg	May 28	May 29	0.57
Average clutch size	6.14	6.17	0.95
Average number of nestlings	5.71	5.58	0.85
Average number of fledglings	4.43	4.92	0.72
*Female body mass (g)*			
Incubation	14.85	14.50	0.26
Chick rearing	12.79	12.51	0.41
*Male body mass (g)*			
Chick rearing	12.37	12.12	0.07

Box occupancy is calculated as the amount of pied flycatcher nests relative to the number of empty boxes at pied flycatcher’s arrival. Adult body masses are determined using information from all breeding pairs in our study site. Average date of first egg excludes control pairs that were not used for the experiment, as well as extra control pairs that were used to complete the cross-fostering. Clutch size and number of nestlings and fledglings consider only those breeding pairs that were included in the experiment, as we did not do any follow-up on the extra pairs on control sites. Significances for differences between control and owl sites were obtained using Wilcoxon rank sum test

### Telomere length assessment

Two months after data collection, the DNA was extracted from whole blood samples using salt extraction alcohol precipitation method (Aljanabi and Martinez 1997). Extracted DNA was diluted in elution buffer BE for DNA preservation and aliquoted. DNA concentration and quality were quantified with Spectrophotometer NanoDrop 2000. Samples were then diluted to concentration of 2.5 ng/µl for subsequent qPCR analysis.

We used quantitative PCR method to measure relative telomere length: the amount of telomeric sequence relative to the amount of a control gene (i.e. non-variable copy number gene) sequence, as previously described in birds (Criscuolo et al. 2009). qPCR analysis was performed on a QuantStudio™ 12 K Flex Real-Time PCR System (Thermo Fisher) using 384-well qPCR plates. We used a final reaction volume of 10µL, 5 ng of genomic DNA, 200 nM of forward and reverse primers and SensiFAST SYBR Lo-ROX mix as MasterMix. qPCR conditions were: an initial denaturation (1 cycle of 3 min at 95 °C), 40 cycles with first step of 10 s at 95 °C, second step of 15 s at 58 °C and third step of 10 s at 72 °C, and finally a melting curve analysis. We used Tel 1b as a forward telomere primer (5′-CGGTTTGTTTGGGTTTGGGTTTGGGTTTGGGTTTGGGTT-3′) and Tel 2b as a reverse telomere primer (5′-GGCTTGCCTTACCCTTACCCTTACCCTTACCCTTACCCT-3′). We used GAPDH as a control gene and designed primers based on collared flycatcher (*Ficedula albicollis,* a close relative of the pied flycatcher) genome, as pied flycatcher genome assembly was not available (forward primer 5′-AACCAGCCAAGTACGATGACAT-3′ and reverse primer 5′-CCATCAGCA GCAGCCTTCA-3′). Before qPCR we performed normal PCR with our GAPDH primers and used gel electrophoresis to confirm the amplification of the pied flycatcher gene. In addition, we checked the specificity of our primers by analysing qPCR melting curves and confirming the presence of a single narrow peak.

Samples were analysed in triplicate and distributed on 384-well plates accordingly: (1) samples from cross-fostering pairs on a same plate; (2) both samples from one individual on the same plate; and (3) if possible, samples from individuals from the same site on the same plate. In the end, the samples were distributed on six different plates. All plates included three internal standards (= same sample on every plate) and one negative control. We used a LinRegPCR version 2017.1 (Ruijter et al. 2009) to determine the baseline fluorescence, the qPCR efficiencies of each reaction (average ± SE efficiencies for telomere and GAPDH reactions were 1.922 ± 0.008 and 1.904 ± 0.003, respectively) and the quantification cycle (*C*_q_) values. Relative telomere length (*T*/*S* ratio, hereafter telomere length) was calculated based on plate-specific efficiencies using the mathematical model presented in Pfaffl (2001). The average intra-plate coefficient of variation (CV ± SE) for *T*/*S* ratio was 11.5 ± 0.56%. The average inter-plate CV based on the three internal standards was 6.9 ± 2.9%. The technical repeatability of triplicate telomere lengths was 0.865 (95% Cl: 0.837–0.886, *p* < 0.001). Chromosomes contain terminal and interstitial telomeric sequences (ITS), but only terminal sequences shorten with age. The qPCR measures both sequences, which can decrease considerably statistical power when studying terminal telomeres due to between-individual differences in ITS amount (Foote et al. 2013). To ensure the validity of our qPCR protocol, we therefore analysed 13 individuals (10 chicks and 3 adults) with both in-gel TRF (i.e. measuring only terminal telomeres) and qPCR. We found a strong correlation between methods (*r* = 0.74, *p* = 0.004, see Online Resource 1), thereby validating the use of qPCR in the pied flycatcher.

### Statistical analyses

We used general linear models and general linear mixed models to examine the effect of predation threat on telomere dynamics in adults and their chicks. As we only had one telomere measurement for parent males at chick rearing, we started by fitting a model including all the adults sampled at chick-rearing stage (males and females with final measurement, *n* = 35), with telomere length as the dependent variable and predator presence (owl or control), sex and their interaction as fixed effects. Because this dataset contained breeding pairs, nest identity was included as a random effect to control for possible similarities in telomere length caused by sharing the same nest. To investigate the rate of telomere change (i.e. telomere length at chick rearing minus telomere length at incubation) in females (*n* = 18), we fitted a model with predator presence (control or owl) and initial telomere length as fixed effects. To avoid statistical artefacts due to regression to the mean phenomenon, the ‘telomere change’ values were corrected using the equation by Verhulst et al. (2013). As control-site females seemed to be consistently a little heavier than owl-site females, we assessed this further by running a repeated-measures model with female body mass as dependent variable and breeding stage, predator presence and their interaction as independent variables.

To test the effects of prenatal and/or early, as well as later parental and environmental components on nestling telomere length (244 measurements from 136 chicks from 24 nests, 12 from control and 12 from owl sites), we fitted a repeated-measures model with, as fixed factors, nestling age (5 or 12 days), predator presence at original nest (control or owl), predator presence at rearing nest (control or owl), their interaction (to address origin-specific responses to the cross-fostering treatment), and the interactions between nestling age and predator presence at original nest as well as nestling age and predator presence at rearing nest. We also used cross-fostering pair (duplicate), and both original and rearing nest box nested within duplicate as random effects. The original nest includes all genetic and prenatal effects as well as early parental effects, while rearing nest includes post-swapping parental effects. The term duplicate reflects variation of nestling traits related to differences between nest pairs such as time of season (Norte et al. 2009). Compound symmetry was used as a covariance structure to model the repeated measures within chicks. We used a similar approach for testing the effect of predation risk on nestling growth rate (i.e. body mass measured at 5 and 12 days). As telomere measurements may vary slightly between different qPCR plates, we tested this ‘plate effect’ by adding plate ID as a random effect to all the telomere models. Ultimately, plate ID was removed from the models as it did not have any obvious effect on the results.

The models were estimated using restricted maximum-likelihood (REML) and Kenward–Roger method was used to calculate degrees of freedom of fixed factors and assess parameter estimates and their standard errors. Normality and heteroscedasticity assumptions were checked visually from the model residuals and deemed satisfactory. Statistical analyses were conducted with SAS statistical software version 9.4 (SAS Institute, Cary, NC, USA).

## Results

**Fig. 1:**
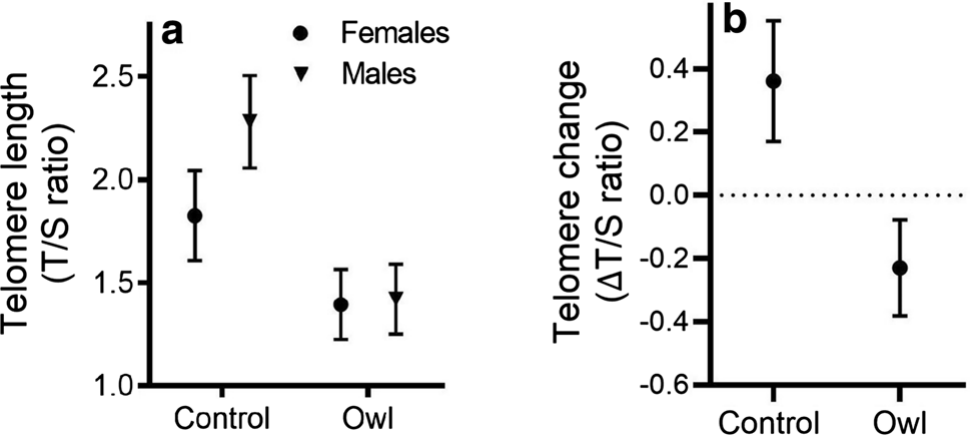
**a** Estimated marginal means (± SE) for telomere length during the chick-rearing phase of female and male parent pied flycatchers nesting either in predator presence (black) or at control sites (grey). **b** Estimated marginal means (± SE) for telomere change between incubation and chick rearing in female pied flycatchers nesting either in predator presence (black) or at control sites (grey)

Adult females and males breeding at owl sites had significantly shorter telomeres at the end of the chick-rearing stage than adults at control sites, and the nearly significant predator presence-by-sex interaction indicates that the differences in telomere length between owl- and control-site individuals could be more pronounced in males than in females (predator presence *F*_1,17.3_ = 6.47, *p* = 0.02; sex *F*_1,14.79_ = 8.45, *p* = 0.01; predator presence × sex *F*_1, 14.79_ = 4.50, *p* = 0.05; Fig. 1a). There was no apparent difference in telomere length between the two groups of females at incubation (*t* test:* df* = 16, *t* = − 0.56, *p* = 0.59), indicating that there were no original quality differences between them in terms of telomere length. However, females inhabiting owl sites exhibited higher rates of telomere shortening between incubation and chick rearing than females inhabiting control sites, which actually tended to show telomere elongation (predator presence *F*_1, 15_ = 5.75, *p* = 0.03; initial telomere length *F*_1, 15_ = 2.24, *p* = 0.16; Fig. 1b). We also ran a repeated-measures model for female telomere length that included main factors breeding stage and predator presence and their interaction, which led to the same conclusions (see Table 1 in Online Resource 2). To further assess possible original quality differences between owl- and control-site adults, we compared their structural body size as measured by wing length and found no differences in either females or males (females, *t* test: *df* = 36, *t* = − 0.97, *p* = 0.34; males, *t* test: *df* 15, *t* = 1.47, *p* = 0.16). As expected, females were heavier during incubation than during chick rearing. Control females were heavier than owl-site females, but there was no significant breeding stage-by-predator presence interaction, which indicates that there was no significant difference in body mass change between females from different sites (Online Resource 2, Table 2).

**Table 2:** Results of a repeated-measures linear mixed model explaining the variability in chick telomere length in relation to age (5d and 12d) and predator presence at both the original and rearing nest sites (control or owl)

Independent variable	Telomere length			
	Estimate ± SE	*df*_num,dem_	*F*/*χ*^2^*	*P*
*Fixed effects*				
Intercept	1.839 ± 0.132			
Age (5d)	1.342 ± 0.139	1, 116.5	230.57	< 0.0001
Original site (control)	0.036 ± 0.131	1, 10.21	0.06	0.806
Rearing site (control)	− 0.378 ± 0.139	1, 11.4	19.41	0.001
Rearing site × age	− 0.081 ± 0.160	1, 116.1	0.26	0.614
Original site × age	− 0.173 ± 0.158	1, 116.5	1.21	0.274
Original site × rearing site	1.140 ± 0.145	1, 93.92	0.93	0.338
*Random effects*				
Original nest (duplicate)	0.001 ± 0.015	1	0.01	0.463
Rearing nest (duplicate)	0.005 ± 0.005	1	0.14	0.354
Duplicate	0.073 ± 0.034	1	6.68	0.005
*Repeated effect*				
Compound symmetry	− 0.035 ± 0.038			
Residual	0.375 ± 0.052			

*F tests were used for significance tests of fixed effects and likelihood ratio tests (*χ*^2^) with mixture distributions and one-sided *p* values were used for random effects

**Fig. 2:**
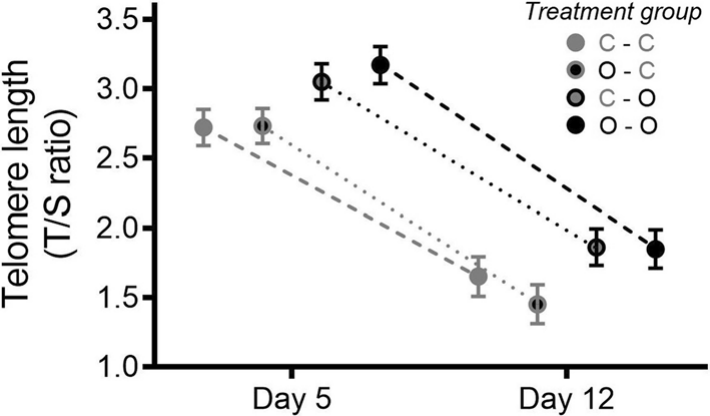
Estimated marginal means (± SE) for telomere lengths of chicks at 5 and 12 days old in each treatment group (C–C: hatched and reared in control site; O–C: hatched in owl site and reared in control site; C–O: hatched in control and reared in owl site; O–O: hatched and reared in owl site; C–O and O–C included the cross-fostered chicks, while C–C and O–O included the chicks that remained in their natal nest). Dotted lines indicate the change in telomere length. Symbols within time points are adjacent to each other for clarification

The telomere length of the chicks shortened significantly between days 5 and 12, but this shortening was not significantly related to predation risk (Table 2, Fig. 2). However, chicks reared at owl sites had consistently longer telomeres during the growth period than chicks reared at control sites, regardless of whether they originated from owl or control sites (Table 2; Fig. 2). Additionally, the growth rate did not differ between cross-fostered groups or according to predator presence at original or rearing nest (see Table in Online Resource 3).

## Discussion

Even though predators are common stressors in the wild and known to have long-term effects on prey physiology and demography (Boonstra 2013; Clinchy et al. 2013), to our knowledge the indirect consequences of predator presence on individual telomere biology have not been evaluated before. Our results from a wild population of birds facing real predation risk indicate that predators may cause long-term costs in terms of telomere length to their near-living prey. We observed that both male and female parent pied flycatchers nesting near predators (breeding pygmy owls) had shorter telomeres at the end of the chick-rearing period than those nesting at control sites. Moreover, females nesting at owl sites suffered from impaired telomere maintenance during breeding compared to females nesting at control sites. While these results for the parents are correlative because the parents were not randomly allocated to the different environments, they provide the first evidence for a potential predator effect on telomere dynamics that should be verified with manipulative experiments. However, we found no evidence for the hypothesis that predator presence would accelerate telomere shortening in nestlings. Instead, chicks reared at owl sites had consistently longer telomeres during the growth period from day 5 to 12. This suggests that the parents are able to buffer the growth of the chicks against the potential stress caused by predator presence.

### Telomere dynamics in parent flycatchers under predation risk

Stress exposure has previously been associated with increased telomere shortening in several species, from human to laboratory and wild animals (Epel et al. 2004; Kotrschal et al. 2007; Herborn et al. 2014; Meillère et al. 2015). In our study, shorter telomeres of pied flycatcher adults nesting at owl sites and the increased telomere shortening in owl-site females may be caused by an increase in glucocorticoids and resulting oxidative stress that arises from the fear and stress of being predated (Angelier et al. 2017). This is supported by the study of Thomson et al. (2010) showing that the levels of stress proteins in blood decreased linearly with the increasing distance to a predator (sparrowhawk *Accipiter nisus*) nest in the pied flycatcher. Additionally, glucocorticoids have been shown to inhibit telomerase activity (Choi et al. 2008), which could explain the difference in telomere dynamics observed in females between owl and control sites. Indeed, while owl-site females tended to lose telomere length between incubation and chick rearing, females breeding at control sites tended to increase their telomere length. While telomere elongation has been documented in other bird species before (Spurgin et al. 2017), this remains controversial and should be further explored in the future by measuring telomerase activity. Alternatively, telomere lengthening in control-site females could be linked to renewal of blood cells following the first blood sampling. Owl-site females could have less resource to renew their blood cells, which could explain why they show telomere shortening while the telomeres of control-site females show elongation.

A recent hypothesis also suggests that telomere shortening may increase during times of substantially increased energy demands due to specific metabolic adjustments (Casagrande and Hau 2019). For instance, a study on humans found that individuals with high physical activity had shorter telomeres than individuals with moderate physical activity (Ludlow et al. 2008). Pied flycatcher parents have been shown to visit their nests more often under increased predation risk (Hakkarainen et al. 2002; Thomson et al. 2010). The fact that pied flycatcher females at owl sites are lighter than at control sites could be suggestive that they have higher activity levels and, thus, higher energy demands. Therefore, this might contribute to explain the difference observed in telomere dynamics between control- and owl-site females. Although there were no differences in female body mass change between control and owl sites, it is likely that higher activity levels could be induced by predator avoidance even before breeding, but that effects on telomere length might only become visible later on since most of the telomere shortening occurs during the following cellular replication.

We measured male telomere length only once. Therefore, we cannot say with certainty whether the change in telomere dynamics would be the same in males as in females. Nevertheless, similar to the females, males at owl sites had significantly shorter telomere length at the end of chick rearing than control males, which could be the result of faster telomere attrition in owl-site males. However, we cannot exclude the possibility that males in owl sites had already shorter telomeres at the beginning of the breeding season, and that this difference persisted through the study period. It has been shown that pied flycatchers avoid breeding in sites inhabited by a pygmy owl (Morosinotto et al. 2010). Consequently, it is possible that only poor-quality males would have been forced to settle at owl sites, since individuals of good quality may be better in competing over territories, and poor-quality individuals may have initially shorter telomeres than individuals of good quality (Le Vaillant et al. 2015). A potential original quality difference cannot be ruled out for females either. There was however no difference in initial (i.e. during incubation) telomere length or in the change in body mass between incubation and chick rearing between owl- and control-site females. Furthermore, females at control and owl sites had similar clutch size, brood size and managed to raise similar number of fledglings. This data would suggest an absence of difference in quality (at least in terms of breeding performance) for the females breeding at control vs. owl sites. Therefore, in case of an original quality difference between individuals choosing to nest in owl or control sites (in terms of telomere length), it would be sex specific and only concern males. We further attempted to examine for potential quality differences by examining the size of the birds at owl and control sites. At least in pied flycatchers (Potti 1998) and 18 species of Parulidae warblers (Francis and Cooke 1986), males with longer wings have reported to arrive earlier at the breeding grounds, and in other studies early arrival has been linked to potentially better individual quality (Lundberg and Alatalo 1992; Saino et al. 1997; Siitari and Huhta 2002; Smith and Moore 2005; but see Sirkiä and Laaksonen 2009). We did not, however, find any differences in wing length between birds at owl and control sites. Furthermore, in this study, the very first arriving (= first breeding) birds were not included, as there were no matching hatching dates in the owl sites to perform the cross-fostering, thus levelling some possible quality differences between birds settling in owl or control sites.

### Telomere dynamics in chicks

Telomere shortening is fastest during the growth stage when cell proliferation is high (Spurgin et al. 2017) and accordingly we found a strong reduction in chick telomere length between days 5 and 12. Contrary to our predictions, we observed consistently longer telomeres in chicks reared at owl sites, while there was no significant effect of the site of origin. This suggests that prenatal and early post-natal effects of predator presence (e.g. through transfer of maternal stress hormone) had little or no importance for telomeres, while later post-natal conditions (i.e. after cross-fostering) were more important. Unexpectedly, predator presence during rearing seems to be positive in terms of chick telomere length.

The lack of a prenatal effect was unexpected because stressed females can transfer stress hormones to their developing young, leading to offspring with increased glucocorticoid levels (Saino et al. 2005; Sheriff et al. 2010) and shorter telomeres (Haussmann and Heidinger 2015). In our study, it is possible that there were no differences in maternal glucocorticoid levels between eggs at owl and control sites, or alternatively that the increase of glucocorticoids in the egg was too minor to cause deleterious effects on telomeres. The unexpected apparent positive effect of predator presence on chick telomere length during rearing may be explained by parental behavioural response to the predator threat. Pied flycatcher parents that experience frequent predator encounters resume feeding nestlings quicker than those being less exposed to predators (Thomson et al. 2011) and both Thomson et al. (2010) and Hakkarainen et al. (2002) have reported increased nest visitation and provisioning rates under increased predation risk in pied flycatchers, contrary to what has been found in some other studies (Tilgar et al. 2011; Zanette et al. 2011). Nevertheless, we did not find differences in growth rate between chicks reared at owl and control sites. The potential extra food received by owl-site chicks could have been used for promoting self-maintenance processes (e.g. antioxidant defenses and telomere length maintenance), or parents may have reduced prey load size (Martindale 1982). Carrying food more often to nest could prevent the chicks from begging, which would reduce nest conspicuousness and, although the entrance hole of our nest boxes is too small for the owl to enter, parents may not perceive their chicks being safe as in old natural cavities owls may access the holes by making them larger (Hakkarainen et al. 2002; Thomson et al. 2010). Begging carries an oxidative cost (Moreno-Rueda et al. 2012), thus any reduced begging activity could also contribute to explain the longer telomeres we observe in chicks raised in owl sites. Additionally, chicks that are exposed to nest predator calls can lower their baseline glucocorticoid levels (Ibáñez-Álamo et al. 2011). High glucocorticoid levels are associated with increased begging rate (Loiseau et al. 2008). Thus, down-regulating glucocorticoids when perceived nest predation risk is high could be adaptive to reduce begging and nest conspicuousness and could contribute to explain our results for chick telomeres. However, gathering data on provisioning rate, prey load size, begging rate and glucocorticoid levels is needed in the future to test these hypotheses.

In conclusion, our study demonstrates that predator presence may affect the telomere length and dynamics of their prey, which could have long-term consequences for the individual in terms of survival probability and add a new hypothesis of how predators may indirectly influence prey demography. While the effects of predation risk seem deleterious in adult birds, the effects seen for nestling telomere length during early rearing were positive, therefore suggesting that different life history stages can be differently affected by increased predation risk. Our results provide further indication for the link connecting environmental stress to cellular/organismal ageing (Angelier et al. 2017), and highlight the potential importance of indirect predator effects on prey physiology for population dynamics.

## Electronic supplementary material

Below is the link to the electronic supplementary material.

Supplementary material 1 (PDF 698 kb)

Supplementary material 2 (PDF 449 kb)

Supplementary material 3 (PDF 449 kb)

Supplementary material 4 (XLSX 38 kb)
