# Supplementary material for: Impact of continuous predator threat on telomere dynamics in parent and nestling pied flycatchers
Source: Oecologia. 2019 Oct 14;191(4):757–66. doi: 10.1007/s00442-019-04529-3 (PMC6853860; doi:10.1007/s00442-019-04529-3)
Supplement: Supplementary file 1 — Supplementary material 1 (PDF 698 kb) [file 442_2019_4529_MOESM1_ESM.pdf]

Electronic Supplementary Material (ESM1)

Impact of continuous predator threat on telomere dynamics in parent and nestling pied flycatchers (Journal: Oecologia)

Tiia Kärkkäinen[a]*, Pauliina Teerikorpi[a], Bineet Panda[b], Samuli Helle[a], Antoine Stier[a,c], Toni Laaksonen[a,d]

[a]Department of Biology, Section of Ecology, University of Turku, Finland

[b]Department of Biology, Section of Genetics and Physiology, University of Turku, Finland

[c]Institute of Biodiversity, Animal Health and Comparative Medicine, University of Glasgow, Glasgow, UK

[d]Natural Resources Institute Finland (LUKE)

*Corresponding author: tmakark@gmail.com

Validation of the qPCR methodology using *in-gel* TRF

In order to validate the qPCR method for use in European pied flycatcher, we analysed n = 13 samples using *in-gel* TRF following a published protocol that has been used successfully in numerous avian species (Haussmann et al. 2012; Tricola et al. 2018). In brief, DNA was extracted from 10 µL of whole blood using the Gentra Puregene Tissue Kit (Qiagen) and kept frozen at -80°C until analysis. Digestion with restriction enzymes was then conducted on 10µg of DNA using *Hae III* (75U), *Hinf I* (15U), and *Rsa I* (40U) in 1X CutSmart enzyme-buffer and incubated overnight at 37°C. DNA samples and ^{32}P labelled size ladder (2-40kb, 1 kb DNA Extension Ladder Invitrogen) were then loaded into a 0.8% non-denaturing agarose gel. DNA was separated using pulse-field gel electrophoresis (14°C at 3 V cm^{-1}, initial switch time 0.5 seconds, final switch time 7.0 seconds) for 19 hours, followed by in-gel hybridization overnight with the ^{32}P γ-ATP probe (5'-CCCTAA-3')$_4$. Hybridized gels were placed on a phosphor screen for 48 hours, which were subsequently scanned with a Typhoon Variable Mode Imager (Amersham Biosciences). Finally, average telomere length was quantified by densitometry in the program ImageJ (version 2.0) within the limits of our molecular size markers (2-40kb). All samples were run using a single gel and intra-gel CV based on duplicates was 2.73 ± 0.30%. The same DNA samples were also analysed with qPCR in a single plate following the methodology described in the main text (intra-plate CV = 7.42 ± 1.34%).

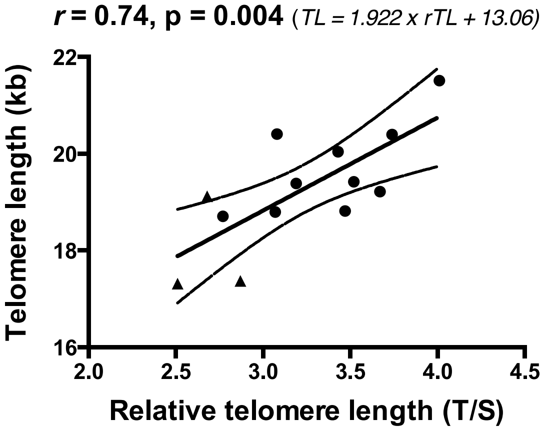

Figure S1: **Correlation between relative telomere length (rTL) measured using qPCR and absolute telomere length (TL) measured using *in-gel* TRF.** We use samples from 10 chicks (circles) and 3 adults (triangles).

Haussmann, M.F., Longenecker, A.S., Marchetto, N.M., Juliano, S.A. & Bowden, R.M. (2012) Embryonic exposure to corticosterone modifies the juvenile stress response, oxidative stress and telomere length. *Proc R Soc B*, **279**, 1447–1456.

Tricola, G.M. et al. (2018) The rate of telomere loss is related to maximum lifespan in birds. *Phil Trans R Soc B*, **373**, 20160445–11.
