# Supplementary material for: Impact of continuous predator threat on telomere dynamics in parent and nestling pied flycatchers
Source: Oecologia. 2019 Oct 14;191(4):757–66. doi: 10.1007/s00442-019-04529-3 (PMC6853860; doi:10.1007/s00442-019-04529-3)
Supplement: Supplementary file 2 — Supplementary material 2 (PDF 449 kb) [file 442_2019_4529_MOESM2_ESM.pdf]

Electronic Supplementary Material (ESM2)

Impact of continuous predator threat on telomere dynamics in parent and nestling pied flycatchers (Journal: Oecologia)

Tiia Kärkkäinen[a]*, Pauliina Teerikorpi[a], Bineet Panda[b], Samuli Helle[a], Antoine Stier [a,c], Toni Laaksonen[a,d]
[a]Department of Biology, Section of Ecology, University of Turku, Finland
[b]Department of Biology, Section of Genetics and Physiology, University of Turku, Finland
[c]Institute of Biodiversity, Animal Health and Comparative Medicine, University of Glasgow, Glasgow, UK
[d]Natural Resources Institute Finland (LUKE)
*Corresponding author: tmakark@gmail.com

Results for the model testing the effect of predation risk on female telomere change

Table 1. Results of repeated-measures linear mixed model explaining the variability in female telomere length in relation to breeding stage (Incubation and Chick rearing) and predator presence (Control or Owl).

	Female telomere length			
Independent variable	Estimate ± se	$df_{num,dem}$	F	*P*
Fixed effects				
Intercept	1.321 ± 0.146	28.88		
Breeding stage (Incubation)	0.242 ± 0.148	1, 16	0.24	0.630
Predator presence (Control)	0.504 ± 0.234	1, 16	1.03	0.324
Breeding stage × Predator presence	-0.599 ± 0.237	1, 16	6.41	0.022
Repeated effect				
Compound symmetry (ID)	0			
Residual	0.120 ± 0.042			

Results for the model testing the effect of predation risk on female mass change

Table 2. Results of repeated-measures linear mixed model explaining the variability in female mass in relation to breeding stage (Incubation and Chick rearing) and predator presence (Control or Owl).

	Female mass			
Independent variable	Estimate ± se	$df_{num,dem}$	F	*P*
Fixed effects				
Intercept	12.509 ± 0.182	25.59		
Breeding stage (Incubation)	1.909 ± 0.217	1, 16	160.62	<.0001
Predator presence (Control)	0.277 ± 0.291	1, 16	6.01	0.026
Breeding stage × Predator presence	0.591 ± 0.348	1, 16	2.88	0.109
Repeated effect				
Compound symmetry (ID)	0			
Residual	0.259 ± 0.092			
