# Supplementary material for: Impact of continuous predator threat on telomere dynamics in parent and nestling pied flycatchers
Source: Oecologia. 2019 Oct 14;191(4):757–66. doi: 10.1007/s00442-019-04529-3 (PMC6853860; doi:10.1007/s00442-019-04529-3)
Supplement: Supplementary file 3 — Supplementary material 3 (PDF 449 kb) [file 442_2019_4529_MOESM3_ESM.pdf]

Electronic Supplementary Material (ESM3)

Impact of continuous predator threat on telomere dynamics in parent and nestling pied flycatchers (Journal: Oecologia)

Tiia Kärkkäinen[a]*, Pauliina Teerikorpi[a], Bineet Panda[b], Samuli Helle[a], Antoine Stier [a,c], Toni Laaksonen[a,d]

[a]Department of Biology, Section of Ecology, University of Turku, Finland
[b]Department of Biology, Section of Genetics and Physiology, University of Turku, Finland
[c]Institute of Biodiversity, Animal Health and Comparative Medicine, University of Glasgow, Glasgow, UK
[d]Natural Resources Institute Finland (LUKE)
*Corresponding author: tmakark@gmail.com

Results for the model testing the effect of predation risk on nestling growth rate

ESM3 Table. Results of repeated-measures linear mixed model explaining the variability in chick body mass in relation to age (5 and 12 days) and predator presence at both original and rearing site (Control or Owl).

	Body mass			
Independent variable	Estimate ± se	$df_{num,dem}$	F ⁄ χ^2*	*P*
Fixed effects				
Intercept	13.679 ± 0.302	40.7		
Age (5d)	-5.949 ± 0.198	1, 213	2643.85	<.0001
Original site (Control)	0.380 ± 0.301	1, 11.5	2.50	0.141
Rearing site (Control)	-0.668 ± 0.342	1, 10.4	2.60	0.137
Rearing site × Age	0.235 ± 0.229	1, 213	1.05	0.307
Original site × Age	-0.132 ± 0.225	1, 211	0.34	0.558
Original site × Rearing site	0.162 ± 0.240	1, 213	0.45	0.503
Random effects				
Original nest (duplicate)	0.286 ± 0.155	1	18.06	<.0001
Rearing nest (duplicate)	0.420 ± 0.223	1	20.45	<.0001
Duplicate	0.071 ± 0.239	1	0.09	0.381
Repeated effect				
Compound symmetry	0.052 ± 0.084			
Residual	0.756 ± 0.100			

*F-tests were used for significance tests of fixed effects, likelihood ratio tests (χ^2) with mixture distributions and one-sided p-values were used for random effects.
